# Risk factors for anthroponotic cutaneous leishmaniasis in unresponsive and responsive patients in a major focus, southeast of Iran

**DOI:** 10.1371/journal.pone.0192236

**Published:** 2018-02-07

**Authors:** Mehdi Bamorovat, Iraj Sharifi, Mohammad Reza Aflatoonian, Hamid Sharifi, Ali Karamoozian, Fatemeh Sharifi, Ahmad Khosravi, Saeid Hassanzadeh

**Affiliations:** 1 Leishmaniasis Research Center, Kerman University of Medical Sciences, Kerman, Iran; 2 Research Center of Tropical and Infectious Diseases, Kerman University of Medical Sciences, Kerman, Iran; 3 HIV/STI Surveillance Research Center, and WHO Collaborating Center for HIV Surveillance, Institute for Futures Studies in Health, Kerman University of Medical Sciences, Kerman, Iran; 4 Research Center for Modeling in Health, Institute for Futures Studies in Health, Kerman University of Medical Sciences, Kerman, Iran; 5 Pharmaceutics Research Center, Institute of Neuropharmacology, Kerman University of Medical Sciences, Kerman, Iran; 6 Department of Pathobiology, Faculty of Veterinary Medicine, Shahid Bahonar University of Kerman, Kerman, Iran; Universita degli Studi di Parma, ITALY

## Abstract

Cutaneous leishmaniasis (CL) is a serious health challenge at the global level due to *Leishmania tropica*. This study was conducted to evaluate the risk factors associated with anthroponotic CL (ACL) in unresponsive (patient who does not heal and remains with an active lesion, despite receiving two courses of intra-lesional Glucantime along with cryotherapy and one cycle of systemic Glucantime) and responsive patients in a major focus in southeastern Iran. A case-control study was conducted from April 2015 to October 2016 in the southeast of Iran. Patients were recruited in a major ACL focus from unresponsive and responsive cases. These patients were compared for environmental, clinical, and demographic characteristic factors. Twenty-five risk related factors were analyzed using multivariate logistic regression and backward elimination stepwise models. *P*<0.05 was defined to be statistically significant. In general, 340 patients with ACL comprising 72 (21.2%) unresponsive cases and 268 (78.8%) responsive cases with active lesions or scars were analyzed by estimating odds ratio (OR). All isolates from 15 responsive and 15 unresponsive patients were characterized as *Leishmania tropica* based on the BLAST and phylogenic analyses by PCR sequences of the Hsp70 and ITS1 loci. Among the 25 variables, 4 major risk factors including poor interior housing conditions (OR = 1.99, confidence interval (CI) = 1–3.93, *P*<0.04), history of chronic diseases (OR = 6.22, CI = 2.51–15.44, *P*≤0.001), duration of lesion in the patients referred ≥13 months (OR = 74.99, CI = 17.24–326.17, *P*≤0.001), and 5–12 months (OR = 7.42, CI = 3.07–17.92, *P*≤0.001) than lesions with ≤4 months of age and age groups ≥51 years (OR = 3.85, CI = 1.04–14.22, *P*<0.04) than those ≤7 years, were significantly associated with unresponsive forms. Improving interior house construction protecting high risk individuals and those with debilitating diseases from being bitten by sand flies, together with the early detection and effective treatment of older age groups with history of chronic diseases are highly important measures for preventing unresponsive forms in patients with ACL in southeastern Iran.

## Introduction

Leishmaniasis imposes a major health impact all over the tropical and subtropical regions of the world [1]. An estimated 0.9 to 1.3 million new cases and 20000 to 30000 deaths occur annually in the world [2]. Cutaneous leishmaniasis (CL) is the most common form of the disease which constitutes approximately 75% of the global cases. Anthroponotic cutaneous leishmaniasis (ACL) caused by *Leishmania tropica* and zoonotic cutaneous leishmaniasis (ZCL) caused by *Leishmania major* are endemic in many parts of Iran with high outbreak rates [3–6]. Therefore, CL has recently become an important health issue in the country. Recently, its incidence has risen considerably and is expanding to new foci [7].

Poverty-stricken individuals are commonly affected by this disease due to their malnutrition, nomadic life, feint immune system, and lack of living standards [2]. The population at the risk of CL in countries with high burden varies between 14 and 100%. It means that approximately 399 million people are subjected to the risk of CL. The highest CL incidence has been reported in Syria with 22.74 per 10000 people, while in Iran it is 2.68 per 10000 people. Based on the recent statistics in CL-high-burden countries, the cases have tripled over the past two decades [8]. ACL has been reported for many years and can be considered as a classic form of the disease in Iran [1]. In urban places, located at higher altitudes, CL is caused by *L*. *tropica* which is spread in an anthroponotic cycle from human to human by female sand fly bites, *Phlebotomus sergenti* [3]. The disease is associated with socioeconomic, religious, cultural, demographic, and environmental factors. Migration of laborers from rural to urban areas for occupational reasons remains a significant risk determinant as well as civil unrest and climate changes for ACL [9].

In Iran, meglumine antimoniate (Glucantime) is the first-line treatment for CL and visceral leishmaniasis (VL), either systemically alone or intra-lesionally along with cryotherapy [10,11]. Patients with CL lesions not responding to pentavalent antimonials treatment have been reported [7]. These patients certainly need effective alternative treatment modalities. Although the disease is a treatable non-healing forms of CL which frequently occur [10]. The treatment of CL is also related to several factors such as geographic modification (environmental factors), type and clinical status of disease, and parasite species [2]. The objective of this study was to investigate the role of environmental, clinical, and demographic characteristic risk factors in creating unresponsive forms when compared with responsive group among patients with ACL in a major focus in southeastern Iran. To our knowledge, the present work is the first study on associated risk-related factors among unresponsive and responsive patients at a national and international level.

## Materials and methods

### Study design

A case-control study was conducted from April 2015 to October 2016 from 10 high risk zones of Kerman district, in the southeast of Iran.

### Ethical consideration

Ethical approval was granted by the joint Ethical Committees of Kerman University of Medical Sciences and Kerman Leishmaniasis Research Center (ethic no. IR.KMU.REC.1394.115, contract no. 94.621). Initially, several face-to-face meetings and interviews with the volunteers and community authorities were organized. In the meetings, the purposes of the study, procedure, and potential benefits were described. Accordingly, patients with CL were treated free of charge with the correct drugs. Those who were suspected of having other underlying diseases were referred to provincial hospitals for further follow-up examinations. Patients with CL willingly participated in the survey. For each patient, a written informed consent form was completed. All demographic and clinical data were kept confidential. Moreover, parents/guardians provided informed consent “on behalf" of all the children that participated.

### Study site

Kerman province is located in southeast Iran, in the southwest of the Kavir-e Lut. This province falls into the arid and semi-arid zones and suffers from scarcity of water, conditions like much of the Iranian plateau. The average annual rainfall is low and decreases toward the southeast, although the topography gives rise to many local variations. Maximum precipitation occurs in winter; the annual average is 142 mm in Kerman. In the province, leishmaniasis is common and also very old. Two major ACL foci are present in the province of Kerman including the Kerman and Bam districts [12].

### Questionnaire

The survey questionnaires were based on the leishmaniasis indicator household questionnaires. Some of the questions of the questionnaire were selected based on previous studies [13,14]. Other questions were selected according to the importance of health issues associated with environmental, demographical, and clinical field of leishmaniasis from the expert’s points of view (S1 Table). Also, the content was evaluated by researchers from different fields (epidemiology, parasitology, and dermatology) in terms of its validity. Before the start of the survey and questioning, several coordinating intra-group meetings were held among the research group to limit the intra evaluation errors. Necessary explanations about the investigation were given to the patients or their parents (guardians). Afterwards, the questioning process proceeded. During the questioning process, the questioner made sure the patients well-understood the questions. Data were collected through house-to-house visits and interviews via structured questionnaires together with direct observation of housing and environmental conditions with permission of the households' heads. The data were accurately recorded by the first author in the questionnaire.

The questionnaire included environmental, clinical, and demographic characteristic risk factors. The 25 risk factors include building, wall, door, window, and interior housing conditions, out of building toilet, presence of small garden, dwelling hygienic condition, number of windows and rooms, presence of domestic animals, dog and manure in home, solid waste management, presence of dogs in close vicinity, duration, number and location of the lesions, treatment conditions, history of chronic diseases, age, sex, education, job, and marital status.

### Unresponsive and responsive participants

**Fig 1:**
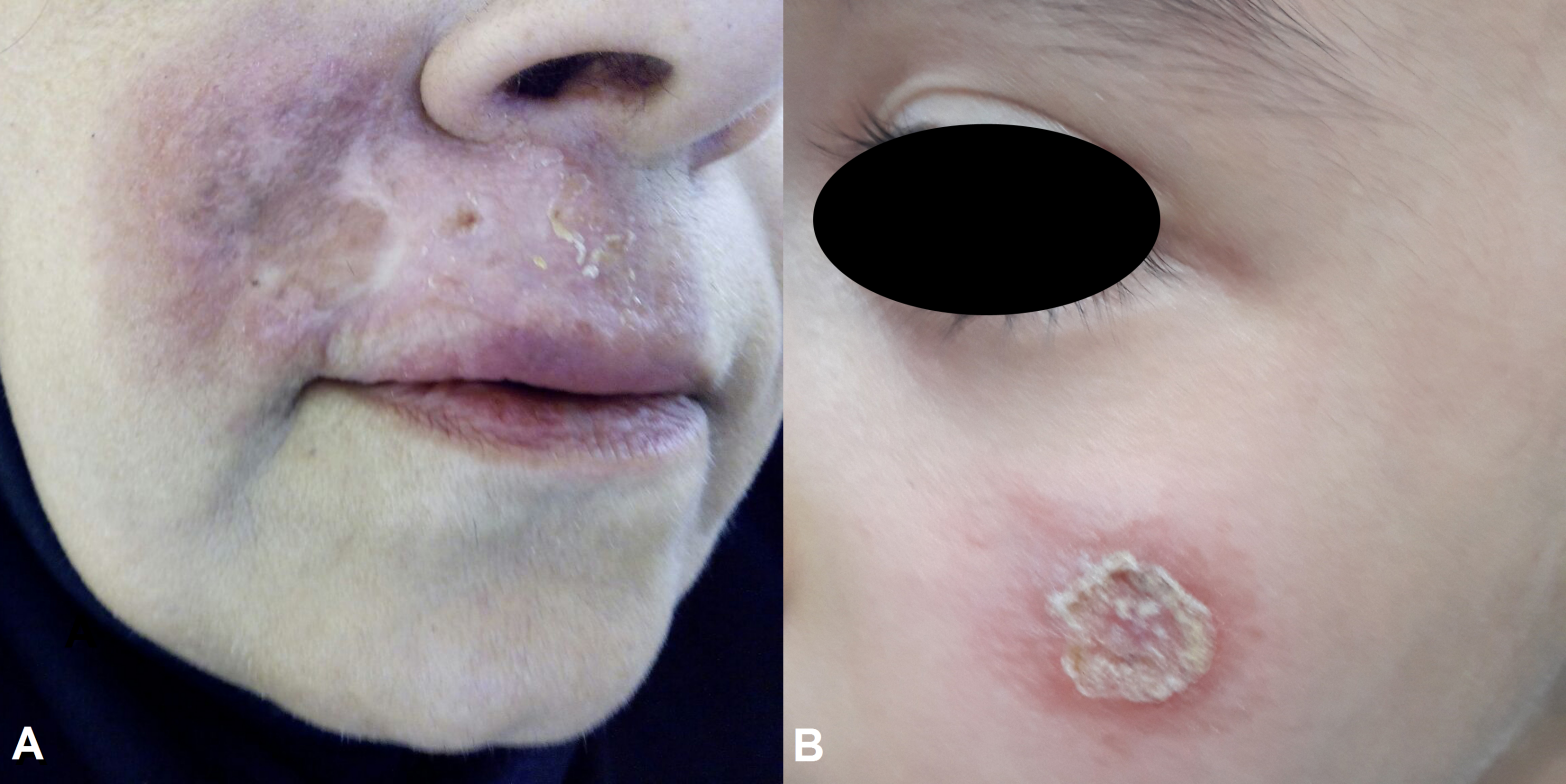
Unresponsive patients with active skin lesions: a 52 years old woman (A) and an 11 years old child (B).

Patients were recruited in a major ACL focus from unresponsive (case) and responsive (control) ACL cases. Unresponsive patient is one who does not heal and remains with an active lesion, despite receiving two courses of intra-lesional Glucantime along with cryotherapy and one cycle of systemic Glucantime (Fig 1). Responsive patient is one whose lesion heals by one treatment cycle with intramuscular administration of Glucantime or intra-lesional Glucantime together with cryotherapy. The patients were compared for their environmental, clinical, and demographic characteristics factors. In total, 340 ACL patients consisting of 72 (21.18%) unresponsive and 268 (78.82%) responsive cases were included in the study.

### Culturing of the organism

A random sub-sampling process was performed to identify the causative agent of CL. Out of the 30 patients in this study, 15 were unresponsive (non-healed) and the remaining were responsive (healed) patients by different foci and month (Table 1). The sampling was performed with the active lesion in both unresponsive and responsive patients. Skin lesions were cleansed with topical antiseptic and aspirated by inserting a 20-gauge needle with a syringe. Promastigotes were grown in Novy-MacNeal-Nicolle (N.N.N) medium at 24±1°C for one week and then sub-cultured in Rosewell Park Memorial Institute (RPMI1640) cell culture medium (Gibco BRL) with 10% inactive fetal calf serum (FCS; Sigma-Aldrich), Penicillin (100 unites/ml), and Streptomicin (100 μg/ml) [15]. Promastigotes were collected by 5 min centrifugation at 8000 rpm and the remaining sample was stored at -20°C for further identification tests.

**Table 1:** The number of isolates that were attempted from unresponsive and responsive cases by different foci and months.

Number of isolates	Unresponsive patients		Responsive patients	
	High risk zones	Date	High risk zones	Date
1	Sarasiab	August 2015	Sarasiab	August 2015
2	Shahrak Pedar	September 2015	Sarbaz St.	September 2015
3	Sarasiab	October 2015	Shahrak Pedar	September 2015
4	Emam St.	October 2015	Emam St.	September 2015
5	Allah abad	October 2015	Allah abad	October 2015
6	Shahrak Sanati	October 2015	Sarasiab	October 2015
7	Sarasiab	November 2015	Shahrak Sanati	October 2015
8	Shahrak Sanati	November 2015	Firozabad	October 2015
9	Firozabad	November 2015	Shahab St.	November 2015
10	Emam St.	November 2015	Sarasiab	November 2015
11	Sarasiab	December 2015	Shahrak Sanati	November 2015
12	Shahrak Seyedi	December 2015	Modiriate St.	November 2015
13	Shahrak Pedar	December 2015	Allah abad	December 2015
14	Shahrak Sanati	January 2016	Shahrak Sanati	December 2015
15	Allah abad	January 2016	Sarasiab	December 2015

### Molecular identification

To identify the parasite species that caused the CL disease in different isolation regions, molecular identification was performed on each cultured sample (Table 2) based on the studies by Schonian et al. (2003) and Van der Auwera et al. (2013) [16,17], with some modifications. DNA was extracted using High Pure PCR Template Purification Kit (Roche, Germany) and it was quantified with a spectrophotometer (NanoDrop-2000c; Thermo Scientific). Partial amplification of *Hsp*70 and *ITS1* was displayed with specific set of primers (Table 3) followed by the PCR amplification conditions in thermal cycler (Flexcycle 2, Analytikjena, Germany). All amplified samples were sequenced for both directions with Microgen Company, Korea. All amplified samples were sequenced, aligned, and edited by Bioedit ver.7.2 [18] and BLAST with online tool (https://blast.ncbi.nlm.nih.gov/Blast.cgi) to show the maximum species homology.

**Table 2:** Distribution of cultured and molecular positivity in uresponsive and responsive pateints.

Unresponsive patients			Responsive patients		
No. cultured	Culture positive No. (%)	Molecular positive No. (%)	No. cultured	Culture positive No. (%)	Molecular positive No. (%)
28	15 (53.6)	*15 (100)	22	15 (62.2)	*15 (100)

*Due to low quantity of extracted DNA based on NanoDrop measurement, three specimens’ extractions were repeated.

**Table 3:** Set of applied primer to amplify candidate loci with optimum PCR conditions.

Assay	Primer name	Primer sequence	Ref	Product (bp)	Annealing	PCR condition (final Concentration)	Cycling protocol	Cycling
*HSP* 70	F25: 5'	GGACGCCGGCACGATTKCT	Van der Auwera et al., 2013	1245–1286	60°	200 μM dNTP’s 0.8 μM of each PCR primer 2 U Taq pol 1,5 mM MgC12	Annealing 60 sec Extension: 120 sec	35
	R1310: 5'	CCTGGTTGTTGTTCAGCCACTC						
*ITS1*-PCR	LITSR: 5'	TGGATCATTTTCCGATG	Schönian et al., 2003	300–350	53°		Annealing 30 sec Extension: 60 sec	
	L5.8S: 5'	TGATACCACTTATCGCACTT						

### Data analysis

The collected data were entered into a computer using SPSS software (version 20.0, SPSS, Inc., Chicago, IL, USA) and analyzed by univariate and multivariate logistic regression tests. *P* value of less than 0.05 was considered statistically significant.

Univariate logistic regression was used to determine if the variables were proper to be used in multivariate logistic regression. Therefore, variables with *p* value less than 0.2 were picked after the analysis. The main reason for using the multivariate method was that the mentioned variables might be confounders. In this way, their effect could be controlled. Then, the backward elimination stepwise method was used to reach the best possible model. Moreover, it is an additional way to exclude the impacts of confounding variables.

## Results

### Molecular finding

Based on the BLAST and phylogenic analyses of PCR sequences, all isolates were characterized as *L*. *tropica* with high percentage pairwise nucleotide similarity in both *Hsp70* (99 to 100%) and *ITS1* (94 to 99%) loci with other WHO reference strains of *L*. *tropica* (S2 Table). Pairwise nucleotide similarity between Kerman isolates and WHO reference strains of *L*. *major* for *Hsp70* and *ITS1* were calculated, 97 to 98% and 84 to 85% based on the BLAST results, respectively. All sequences were submitted in NCBI nucleotide database with GenBank accession numbers KY661706-KY661710.

### Risk factor analysis

Generally, 340 ACL patients comprising 72 (21.2%) unresponsive and 292 (78.8%) responsive cases with active lesions or scars were analyzed for potential environmental (Table 4), clinical (Table 5), and demographical factors (Table 6). According to univariate and multivariate logistic analyses, 4 major variables of the overall risk factors including poor interior housing conditions, longer duration of lesion, patients with history of chronic diseases, and age groups were significantly associated with the non-healing lesions (S1 Data).

**Table 4:** Odds ratios for potential environmental risk factors with anthroponotic cutaneous leishmaniasis in southeastern Iran.

Variable	Unresponsive patients	Responsive patients	OR	Univariate95%CI	p-Value	OR	Multivariate95%CI	p-Value
**Building Condition**								
^1^ Unsuitable	49	163	1.37	0.79–2.38	0.26			
^2^ Suitable	23	105	1					
**WallCondition**								
Unsuitable	55	192	1.28	0.69–2.34	0. 42			
Suitable	17	76	1					
**Door and window Net**								
No	61	199	1.92	0.95–3.86	0.06			
Yes	11	69	1					
**Interior housing condition**								
Unsuitable	34	93	1.68	0.99–2.85	0.05	1.99	1–3.93	0.04
Suitable	38	175	1			1		
**Out of building toilet**								
No	17	85	0.66	0.36–1.21	0.18			
Yes	55	183	1					
**Presence of small garden**								
No	10	45	0.79	0.38–1.67	0.55			
Yes	62	223	1					
**Dwelling hygienic condition**								
Unsuitable	28	121	0.77	0.45–1.31	0.34			
Suitable	44	147	1					
**Presenceof palm and orange trees in home**								
No	67	254	0.73	0.25–2.12	0.57			
Yes	5	14	1					
**Presenceof domestic animal**								
No	38	160	0.75	0.44–1.27	0.29			
Yes	34	108	1					
**Presenceof dog in home**								
No	57	228	0.66	0.34–1.29	0.22			
Yes	15	40	1					
**Presenceof manure in home**								
No	62	231	0.99	0.46–2.1	0.98			
Yes	10	37	1					
**Solid waste management**								
Unsuitable	4	47	0.27	0.09–0.79	0.01			
Suitable	68	221	1					
**Presenceof dog in region**								
No	8	50	0.54	0.24–1.2	0.13			
Yes	64	218	1					
**Number of room**								
≤2	25	121	1.29	0.41–4.03	0.66			
3–5	43	122	2.2	0.72–6.69	0.16			
≥6	4	25	1					
**Number of window**								
≤2	2	2	3.15	0.43–22.98	0.25			
3–4	19	105	0.57	0.31–1.02	0.059			
≥5	51	161	1					

^1^Unsuitable: Denotes the patients who live in unsuitable housing conditions in terms of building, interior housing, wall and dwelling hygienic conditions and solid waste. If in the living places (building, interior housing and wall), there are cracks and crevices, they are considered unsuitable. If there is garbage and sewage in front of or near the home, it will be considered unsuitable dwelling hygienic condition. If in their place of residence, there is solid waste, it will be considered unsuitable.

^2^Suitable: Denotes the patients who live in suitable housing condition in terms of building, interior housing, wall, dwelling hygienic condition and solid waste management. If in the living places (building, interior housing and wall), there are no cracks and crevices, they are considered suitable. If there is no garbage and sewage in front of or near the home, it will be considered suitable dwelling hygienic condition. If in their place of residence, there is solid waste management, it will be considered suitable.

**Table 5:** Odds ratios for potential clinical status risk factors with anthroponotic cutaneous leishmaniasis in southeastern Iran.

Variable	Unresponsive patients	Responsive patients	OR	Univariate95%CI	p-Value	OR	Multivariate95%CI	p-Value
**Location of lesion**								
Hands	25	145	0.66	0.27–1.62	0.37			
Face	38	68	2.16	0.9–5.18	0.08			
Legs	1	24	0.16	0.01–1.38	0.09			
Other	8	31	1					
**Treatmentcondition**								
^1^ Incompletetreatment	16	55	1.1	0.58–2.07	0.75			
^2^ Complete treatment	56	213	1					
**History of chronic disease**								
Yes	22	26	4.09	2.15–7.8	≤0.001	6.22	2.51–15.44	≤0.001
No	50	242	1			1		
**Duration of lesion (month)**								
≤4	7	141	1			1		
5–12	47	123	7.69	3.35–17.65	≤0.001	7.42	3.07–17.92	≤0.001
≥13	18	4	90.64	24.14–340.26	≤0.001	74.99	17.24–326.17	≤0.001
**Number of lesion**								
≤2	65	225	1.77	0.76–4.13	0.18			
≥3	7	43	1					

^1^Incomplete treatment: The patients who did not receive a course of intramuscular (IM) treatment (20 mg/kg/daily for 3 weeks) or intralesional (IL) along with cryotherapy (20 mg/kg/weekly for a maximum of 12 weeks).

^2^Completed treatment: The patients who received a course of treatment.

**Table 6:** Odds ratios for potential demographical risk factors with anthroponotic cutaneous leishmaniasis in southeastern Iran.

Variable	Unresponsive patients	Responsive patients	OR	Univariate95%CI	p-Value	OR	Multivariate95%CI	p-Value
**Age (year)**								
≤7	19	65	1			1		
8–15	18	82	0.75	0.36–1.54	0.43	0.65	0.25–1.67	0.37
16–30	13	56	0.79	0.36–1.75	0.56	1.03	0.37–2.87	0.95
31–50	13	49	0.9	0.4–2.01	0.81	0.8	0.28–2.23	0.47
≥51	9	16	1.92	0.73–5.04	0.18	3.85	1.04–14.22	0.04
**Sex**								
Female	30	134	0.71	0.42–1.2	0.21			
Male	42	134	1					
**Education**								
Illiterate	31	78	3.06	1.4–6.67	0.005			
Primary and Secondary education	31	113	2.11	0.97–4.55	0.05			
High school and university education	10	77	1					
**Job**								
Unemployed	6	44	2.16	0.88–5.29	0.09			
Employed	66	224	1					
**Marital status**								
Single	57	178	1.92	1.03–3.58	0.04			
Married	15	90	1					

The odds of unresponsive form in patients with unsuitable interior housing condition were significantly higher than in patients with suitable interior housing condition (OR = 1.99, CI = 1.00–3.93, *P*<0.04). The risk of patients with history of chronic diseases was the major factor which had a more profound impact on unresponsive form when compared with patients having no history of chronic diseases (OR = 6.22, CI = 2.51–15.44, *P*≤0.001). Patients referred ≥13 months following the onset of lesion (OR = 74.99, CI = 17.24–326.17, *P*≤0.001) and 5 to 12-months old (OR = 7.24, CI = 3.07–17.92, *P*≤0.001) had significantly higher odds than those ≤4 months duration of lesion. Similarly, patients aged ≥51 years (OR = 3.85, CI = 1.04–14.22, *P*<0.04) demonstrated significantly higher odds than those ≤7 years. Other risk factors including building, wall, door, and window conditions, out of building toilet, presence of small garden, dwelling hygienic condition, number of windows, rooms, domestic animals, dog, manure in home, solid waste management, presence of dog in close vicinity, number and location of the lesions, treatment condition, sex, education, job, and marital status were not significantly associated with the incidence of non-healing lesions (*P*>0.05).

## Discussion

Cutaneous leishmaniasis is one of the most serious health problems in Iran, especially in Kerman province, southeastern Iran [5,19]. Previous studies have shown that CL in Kerman district was predominantly of ACL type. Similarly, the majority of the cases which occurred in southern districts were ACL [19]. However, in this study, attempt was made to use molecular methods powered by sequencing amplification regions of *Hsp*70 and *ITS1* to identify the *Leishmania* isolates. Nucleotide polymorphism as discriminative molecular marker in these regions is highly recommended [17,20–22]. Based on the method used, all samples were *L*. *tropica*, due to the widespread prevalence of this endemic species in this area.

Human health is significantly affected by housing and environmental conditions [13,23]. In the present study, poor interior housing condition was also identified as a risk factor among unresponsive patients with ACL. The results of the present study showed that creation of unresponsive forms in patients with unsuitable interior housing condition was significantly higher than those with suitable interior housing condition (*P*<0.05). The rate of sand flies in the cracks and crevices of walls and ceilings in unsuitable houses is higher than suitable ones [24,25]. This study showed that there was a positive association between the rate of unresponsiveness in ACL and poor interior housing conditions. The people who live in unsuitable interior housing condition in endemic areas are more susceptible to infection with CL, due to the presence of parasite and suitable condition for propagation of sand fly vectors [24,25]. Generally, these individuals belong to low income and poor social class. Therefore, they provide their needs in difficulty and are always looking for a job. These people in case of infection with CL receive their treatment too late or do it irregularly and disregard the disease consequences. It seems that owing to the prolonged duration of the disease, irregular and untimely treatment, the probability of developing unresponsive forms increases in these individuals.

Efforts are being made to sensitize patients in order to complete treatment. Treatment results being poorly defined make it difficult to report on this indicator [8]. In our investigation, among the patients who had started treatment after a long time or with incomplete treatment, had greater odds than those who started treatment after a short onset of the disease (*P*≤0.001). Due to lack of active-case detection approaches and awareness among the inhabitants, arbitrary treatments, and lack of medical care, the disease was not early detected. Hence, the treatment began at least 4 months thereafter. Therefore, in this group, the treatment procedure was more complicated and difficult. Incomplete or CL unsuccessful treatment is sometimes due to increased drug resistance of the *Leishmania* [26,27].

The pathogenesis of the increased prevalence of infection in patients with the history of chronic diseases such as diabetes and opium addiction is affected by the role of innate immune response. Patients with diabetes mellitus (DM) have been more infected than those without DM. Therefore, the increased prevalence of infections could be due to deficiency in immunity [28,29]. It is noteworthy that opium as an important public health problem is common in southeastern Iran and neighboring countries [12]. A recent study in 2016 showed that CL lesions in opium addicted patients were more severe than the control group in terms of the size, number, and duration of lesions [12]. However, the causes of the difference in severity of the lesions are not yet well recognized. According to Sacerdote et al. (1997) and Wei et al. (2003), a significant immunosuppressive effect could be exerted by opioids including morphine and heroin, derivative of opium alkaloids [30,31]. Also, morphine disturbs phagocytic cell role, natural killer cell function, antibody production, cell-mediated immunity, nitrogen intermediates, and reactive oxygen [32–36]. In the present study, patients with the history of diabetes, opium addiction, hypertension/high blood pressure, cardiovascular problems, and tuberculosis, developed more significantly unresponsive forms than those without history of chronic diseases (*P≤0*.*001*).

In several countries where the number of local people is low or the population is frequently altered, all age groups are affected [37–39]. Also, in Kerman, ACL cases have been reported in all age groups, although children in colder seasons contracted the disease more frequently than the older individuals [40]. The present study showed that unresponsiveness in ACL was significantly associated with the increasing age (*P<0*.*05*). Probably, defect in cellular innate immunity due to various factors such as chronic diseases plays a role in the pathogenesis of the increased unresponsive forms in patients in older age groups. The control of CL is interceded by cellular immune responses. It is worth noting that human life styles alternations, environmental condition, immune responses, treatment failure and drug resistance are potential factors that could affect the prevalence of CL [9,23,41,42].

## Conclusion

In this study, four major risk factors, including interior housing conditions, history of chronic diseases, duration of lesion, and age groups were significantly associated with the development of unresponsive forms. Improving interior house construction, early detection and effective treatment, protecting persons and those with debilitating diseases from being bitten by sand flies, especially in older age groups with the history of chronic diseases are exceedingly important measures for prevention of unresponsive forms of ACL in southeastern Iran. To prevent the disease, it is therefore essential that the national health authorities implement appropriate control strategies.

## Supporting information

S1 Data Output of all analyses in unresponsive and responsive patients. (RAR)

S2 Data SPSS data file. (SAV)

S1 Table The original questionnaire in both English and Persian (Farsi) languages. (DOC)

S2 Table WHO reference strains of *Leishmania tropica* and *Leishmania major*. (DOCX)
