# Supplementary material for: Risk factors for anthroponotic cutaneous leishmaniasis in unresponsive and responsive patients in a major focus, southeast of Iran
Source: PLoS One. 2018 Feb 7;13(2):e0192236. doi: 10.1371/journal.pone.0192236 (PMC5802920; doi:10.1371/journal.pone.0192236)
Supplement: S1 Table — (DOC) [file pone.0192236.s003.doc]

**S1 Table. The original questionnaire in both English and Persian (Farsi) languages.**

**Table 1. Environmental characteristic risk factors**

| Presence of palm and orange tree in home | | Dwelling hygienic condition | | Presence of small garden | | Out of building toilet | | Interior housing condition | | Door and window Net | | Wall condition | | Building condition | | Number |
| --- | --- | --- | --- | --- | --- | --- | --- | --- | --- | --- | --- | --- | --- | --- | --- | --- |
| Yes | NO | Suitable | Unsuitable | Yes | NO | Yes | No | Suitable | Unsuitable | Yes | NO | Suitable | Unsuitable | Suitable | Unsuitable |
|  |  |  |  |  |  |  |  |  |  |  |  |  |  |  |  |  |
|  |  |  |  |  |  |  |  |  |  |  |  |  |  |  |  |  |
|  |  |  |  |  |  |  |  |  |  |  |  |  |  |  |  |  |
|  |  |  |  |  |  |  |  |  |  |  |  |  |  |  |  |  |
|  |  |  |  |  |  |  |  |  |  |  |  |  |  |  |  |  |
|  |  |  |  |  |  |  |  |  |  |  |  |  |  |  |  |  |
|  |  |  |  |  |  |  |  |  |  |  |  |  |  |  |  |  |
|  |  |  |  |  |  |  |  |  |  |  |  |  |  |  |  |  |
|  |  |  |  |  |  |  |  |  |  |  |  |  |  |  |  |  |

| Number of window | Number of room | Presence of dog in region | | Solid waste management | | Presence of manure in home | | Presence of dog in home | | Presence of domestic animal | | Number |
| --- | --- | --- | --- | --- | --- | --- | --- | --- | --- | --- | --- | --- |
|  |  | Yes | No | Suitable | Unsuitable | Yes | No | Yes | No | Yes | No |  |
|  |  |  |  |  |  |  |  |  |  |  |  |  |
|  |  |  |  |  |  |  |  |  |  |  |  |  |
|  |  |  |  |  |  |  |  |  |  |  |  |  |
|  |  |  |  |  |  |  |  |  |  |  |  |  |
|  |  |  |  |  |  |  |  |  |  |  |  |  |
|  |  |  |  |  |  |  |  |  |  |  |  |  |
|  |  |  |  |  |  |  |  |  |  |  |  |  |
|  |  |  |  |  |  |  |  |  |  |  |  |  |
|  |  |  |  |  |  |  |  |  |  |  |  |  |

**Table 2.** Demographic characteristic risk factors

| Marital status | | Job | | Education | | | Sex | | Age(year) | Number |
| --- | --- | --- | --- | --- | --- | --- | --- | --- | --- | --- |
| Married | Single | Unemployed | Employed | High school and university education | Primary and Secondary education | Illiterate | Male | Female |
|  |  |  |  |  |  |  |  |  |  |  |
|  |  |  |  |  |  |  |  |  |  |  |
|  |  |  |  |  |  |  |  |  |  |  |
|  |  |  |  |  |  |  |  |  |  |  |
|  |  |  |  |  |  |  |  |  |  |  |
|  |  |  |  |  |  |  |  |  |  |  |
|  |  |  |  |  |  |  |  |  |  |  |
|  |  |  |  |  |  |  |  |  |  |  |
|  |  |  |  |  |  |  |  |  |  |  |

**Table 3.** **Clinical characteristic risk factors (Unresponsive patients)**

| Unresponsive patients | | | | | | | | | | Number |
| --- | --- | --- | --- | --- | --- | --- | --- | --- | --- | --- |
| Number of lesion | Duration of lesion  (month) | History of chronic diseases | | Treatment condition | | Location of lesion | | | |  |
|  |  | Yes | NO | Complete treatment | Incomplete treatment | Other | Legs | Face | Hands |  |
|  |  |  |  |  |  |  |  |  |  |  |
|  |  |  |  |  |  |  |  |  |  |  |
|  |  |  |  |  |  |  |  |  |  |  |
|  |  |  |  |  |  |  |  |  |  |  |
|  |  |  |  |  |  |  |  |  |  |  |
|  |  |  |  |  |  |  |  |  |  |  |
|  |  |  |  |  |  |  |  |  |  |  |
|  |  |  |  |  |  |  |  |  |  |  |
|  |  |  |  |  |  |  |  |  |  |  |

**Table 3. Clinical characteristic risk factors (Responsive patients)**

| Responsive patients | | | | | | | | | | Number |
| --- | --- | --- | --- | --- | --- | --- | --- | --- | --- | --- |
| Number of lesion | Duration of lesion  (month) | History of chronic diseases | | Treatment condition | | Location of lesion | | | |  |
|  |  | Yes | NO | Complete treatment | Incomplete treatment | Other | Legs | Face | Hands |  |
|  |  |  |  |  |  |  |  |  |  |  |
|  |  |  |  |  |  |  |  |  |  |  |
|  |  |  |  |  |  |  |  |  |  |  |
|  |  |  |  |  |  |  |  |  |  |  |
|  |  |  |  |  |  |  |  |  |  |  |
|  |  |  |  |  |  |  |  |  |  |  |
|  |  |  |  |  |  |  |  |  |  |  |
|  |  |  |  |  |  |  |  |  |  |  |
|  |  |  |  |  |  |  |  |  |  |  |

**پرسشنامه**

**جدول1. ریسک فاکتورهای محیطی**

| شماره | وضعیت نمای ساختمان | | وضعیت نمای دیوارها | | وجود توری پنجره ها و درهای ورودی | | وضعیت معماری و نمای درونی ساختمان | | سرویس بهداشتی خارج از ساختمان | | وجود باغچه | | وضعیت فاضلاب ، آبروها و زباله در جلوی منزل | |
| --- | --- | --- | --- | --- | --- | --- | --- | --- | --- | --- | --- | --- | --- | --- |
|  | نا مناسب | مناسب | نا مناسب | مناسب | بله | خیر | نا مناسب | مناسب | بله | خیر | بله | خیر | نامناسب | مناسب |
|  |  |  |  |  |  |  |  |  |  |  |  |  |  |  |
|  |  |  |  |  |  |  |  |  |  |  |  |  |  |  |
|  |  |  |  |  |  |  |  |  |  |  |  |  |  |  |
|  |  |  |  |  |  |  |  |  |  |  |  |  |  |  |
|  |  |  |  |  |  |  |  |  |  |  |  |  |  |  |
|  |  |  |  |  |  |  |  |  |  |  |  |  |  |  |
|  |  |  |  |  |  |  |  |  |  |  |  |  |  |  |
|  |  |  |  |  |  |  |  |  |  |  |  |  |  |  |
|  |  |  |  |  |  |  |  |  |  |  |  |  |  |  |

| شماره | وجود درخت نخل و نارنج در منزل | | نگهداری حیوانات خانگی در خانه | | نگهداری سگ در خانه | | انبار کود حیوانی در منزل | | وضعیت جمع آوری زباله در محله | | وجود سگهای ولگرد در محله | | تعدا اتاقها | تعداد پنجره ها |
| --- | --- | --- | --- | --- | --- | --- | --- | --- | --- | --- | --- | --- | --- | --- |
|  | بله | خیر | بله | خیر | بله | خیر | بله | خیر | نا مناسب | مناسب | بله | خیر |  |  |
|  |  |  |  |  |  |  |  |  |  |  |  |  |  |  |
|  |  |  |  |  |  |  |  |  |  |  |  |  |  |  |
|  |  |  |  |  |  |  |  |  |  |  |  |  |  |  |
|  |  |  |  |  |  |  |  |  |  |  |  |  |  |  |
|  |  |  |  |  |  |  |  |  |  |  |  |  |  |  |
|  |  |  |  |  |  |  |  |  |  |  |  |  |  |  |
|  |  |  |  |  |  |  |  |  |  |  |  |  |  |  |
|  |  |  |  |  |  |  |  |  |  |  |  |  |  |  |
|  |  |  |  |  |  |  |  |  |  |  |  |  |  |  |

**جدول 2. ریسک فاکتورهای دموگرافیک**

| شماره | سن بر حسب سال | جنس | | تحصیلات | | | شغل | | وضعیت تاهل | |
| --- | --- | --- | --- | --- | --- | --- | --- | --- | --- | --- |
|  |  | مرد | زن | بیسواد | ابتدایی و راهنمایی | دبیزستان و دانشگاهی | کارمند | بیکار | مجرد | متاهل |
|  |  |  |  |  |  |  |  |  |  |  |
|  |  |  |  |  |  |  |  |  |  |  |
|  |  |  |  |  |  |  |  |  |  |  |
|  |  |  |  |  |  |  |  |  |  |  |
|  |  |  |  |  |  |  |  |  |  |  |
|  |  |  |  |  |  |  |  |  |  |  |
|  |  |  |  |  |  |  |  |  |  |  |
|  |  |  |  |  |  |  |  |  |  |  |
|  |  |  |  |  |  |  |  |  |  |  |

**جدول3. ریسک فاکتورهای بالینی بیماران عدم پاسخ به درمان**

| شماره | بیماران عدم پاسخ به درمان | | | | | | | | | |
| --- | --- | --- | --- | --- | --- | --- | --- | --- | --- | --- |
|  | محل ضایعه | | | | شرایط درمان | | سابقه بیماریهای مزمن | | مدت زخم بر حسب ماه | تعداد زخم |
|  | دست | صورت | پا | سایر | درمان ناقص | درمان کامل | خیر | بله |  |  |
|  |  |  |  |  |  |  |  |  |  |  |
|  |  |  |  |  |  |  |  |  |  |  |
|  |  |  |  |  |  |  |  |  |  |  |
|  |  |  |  |  |  |  |  |  |  |  |
|  |  |  |  |  |  |  |  |  |  |  |
|  |  |  |  |  |  |  |  |  |  |  |
|  |  |  |  |  |  |  |  |  |  |  |
|  |  |  |  |  |  |  |  |  |  |  |
|  |  |  |  |  |  |  |  |  |  |  |

**جدول3. ریسک فاکتورهای بالینی بیماران پاسخ به درمان**

| شماره | بیماران پاسخ به درمان | | | | | | | | | |
| --- | --- | --- | --- | --- | --- | --- | --- | --- | --- | --- |
|  | محل ضایعه | | | | شرایط درمان | | سابقه بیماریهای مزمن | | مدت زخم بر حسب ماه | تعداد زخم |
|  | دست | صورت | پا | سایر | درمان ناقص | درمان کامل | خیر | بله |  |  |
|  |  |  |  |  |  |  |  |  |  |  |
|  |  |  |  |  |  |  |  |  |  |  |
|  |  |  |  |  |  |  |  |  |  |  |
|  |  |  |  |  |  |  |  |  |  |  |
|  |  |  |  |  |  |  |  |  |  |  |
|  |  |  |  |  |  |  |  |  |  |  |
|  |  |  |  |  |  |  |  |  |  |  |
|  |  |  |  |  |  |  |  |  |  |  |
|  |  |  |  |  |  |  |  |  |  |  |
