# Supplementary material for: Risk factors for anthroponotic cutaneous leishmaniasis in unresponsive and responsive patients in a major focus, southeast of Iran
Source: PLoS One. 2018 Feb 7;13(2):e0192236. doi: 10.1371/journal.pone.0192236 (PMC5802920; doi:10.1371/journal.pone.0192236)
Supplement: S2 Table — (DOCX) [file pone.0192236.s004.docx]

**S2 Table. WHO reference strains of *L. tropica* and *L. major*.**

**WHO references sequences for both *Hsp70* and *ITS1* loci.**

***Hsp70***

| WHO code | Species | Sequence ID (*Hsp70)* |
| --- | --- | --- |
| MHOM/IL/83/IL24 | *L. major* | HF586381 |
| MHOM/ML/80/LEM155 | *L. major* | HF586382 |
| MHOM/JO/90/JH39 | *L. major* | HF586392 |
| MHOM/DZ/89/LIPA228 | *L. major* | HF586398 |
| MHOM/MA/88/LEM1314 | *L. tropica* | HF586347 |
| MHOM/YE/86/LEM1015 | *L. tropica* | HF586348 |
| MHOM/EG/90/LPN65 | *L. tropica* | HF586405 |
| ISER/MA/89/LEM1694 | *L. tropica* | HF586409 |

***ITS1***

| WHO code | Species | Sequence ID *(ITS1)* |
| --- | --- | --- |
| MHOM/CM/97/MOK1 | *L. major* | HG512952 |
| MHOM/BF/2000/COU5 | *L. major* | HG512954 |
| MHOM/SD/2003/LCB33 | *L. major* | HG512958 |
| MHOM/SN/2004/LCB39 | *L. major* | HG512962 |
| MHOM/YE/86/LEM1015 | *L. tropica* | HG512919 |
| MHOM/MA/88/LEM1314 | *L. tropica* | HG512922 |
| ISER/MA/89/LEM1694 | *L. tropica* | HG512925 |
| MHOM/EG/90/LPN65 | *L. tropica* | HG512927 |
